# A 5-years results of the Ponseti method in the treatment of congenital clubfoot: a retrospective study

**DOI:** 10.1007/s00590-022-03353-5

**Published:** 2022-08-13

**Authors:** Almaw Bitew, Debas Yaregal Melesse, Biruk Adie Admass

**Affiliations:** 1 Department of Orthopaedic Surgery, College of Medicine and Health Sciences, University of Gondar, P.O. Box 196, Gondar, Ethiopia; 2 Department of Anaesthesia, College of Medicine and Health Sciences, University of Gondar, P.O. Box 196, Gondar, Ethiopia

**Keywords:** Clubfoot, Idiopathic, Children, Orthopedic, Ponseti

## Abstract

**Background:**

Clubfoot is a common congenital deformity affecting mobility of children. It leads to pain and disability. The Ponseti treatment method is non-surgical method for the correction of clubfoot. There is variation from country to country in how the result of clubfoot management is measured and reported. This study aimed to assess the 5-years results of the Ponseti method in the treatment of congenital clubfoot which was performed for children aged under 2 years in western Amhara, Ethiopia.

**Objectives:**

The objective of this study was to assess a 5-years results of the Ponseti method in the treatment of congenital clubfoot among under 2-years old children in Felege Hiwot Referral Hospital, Bahir-Dar, Ethiopia, from 2015 to 2019 G.C.

**Methods:**

A facility-based retrospective cross-sectional study was conducted. After receiving letter of ethical clearance from the University, IRB board, the data were collected from medical record/charts of patients’ who received Ponseti treatment, and the collected data were entered and analyzed with Statistical Package for Social Sciences version 21. The results were presented with texts and tables.

**Results:**

A total of 200 children with 256 congenital clubfeet involved in the study. Among the total study subjects, 143 (71.5%) had unilateral clubfeet. The 5-years results of the Ponseti method in the treatment of congenital clubfoot conducted in 200 children with clubfeet were 187 (93.5%) with 95% (CI 90–99.5). Among the study participants, males were 66.5% (almost two times from females).

**Conclusions and recommendations:**

According to the results from a 5-years data showed that the Ponseti method in the treatment of congenital clubfoot was successful with a success rate of 93.5%. We recommend that children with congenital clubfeet should be managed with Ponseti treatment method timely.

## Background

Congenital talipes equinovarus is the commonest congenital anomaly with an incidence of one to two per 1000 live births [1]. It is defined as a fixation of the foot in a hand-like orientation in adduction, supination and varus with concomitant soft tissue abnormalities [2].

The main objective of treatment for congenital clubfoot is to obtain pain free, plantigrade foot, with good mobility and without calluses. In general, children who suffer from such deformation undergo some type of surgery to complete the correction. Currently, manipulation, redresions and casting according to Ponseti method are the ‘‘gold standard,’’ and this method is endorsed by the American Association of Orthopedic Surgeons [3]. The Ponseti method of clubfoot treatment has been established as the gold standard of care in most pediatric orthopedic centers worldwide [4]. Ponseti method is a very effective method to correct the clubfoot deformities [5].

The Ponseti method has dramatically altered the management of clubfoot, with particular implications for limited-resource settings [6]. Accelerated increases compliance as well as reducing treatment and travel costs for parents, more so in developing countries [7]. Ponseti treatment and traditional treatment of clubfoot (talipes equinovarus), the Ponseti method is now accepted as standard treatment for this deformity [8]. Treatment for idiopathic congenital clubfoot by means of the Ponseti method brings better results together with less soft-tissue injury, thus confirming the effectiveness and good reproducibility of this method [9]. Non-compliance with post tenotomy bracing, educational level (less than high school) of parents, inaccess of the parents to the internet were some of the factors that affect outcome after the use of the Ponseti method for the management of idiopathic clubfoot while age and sex of the patients was not found to have a significant effect on the recurrence of the deformity [10]. The initial Pirani score, compliance with the foot abduction brace and the age at the first casting are three independent factors for relapse in clubfoot [11]. Non-compliance and the educational level of the parents (high-school education or less) are significant risk factors for the recurrence of clubfoot deformity after correction with the Ponseti method [12].

The Pirani Score is a simple and reliable system to determine severity and monitor progress in the Assessment and Treatment of Clubfoot [13]. During Ponseti Management of Clubfoot, the Pirani Score Record shows whether the deformity is correcting normally or whether there is a problem, and the degree of correction of each component of the clubfoot. The Pirani Score is also utilized to assist in determining when to perform the Tenotomy [14]. A Pirani Scoring 4 or more is likely to require at least four casts, and one scoring less than 4 will require three or fewer [15]. A foot with a hindfoot score of 2.5 or 3 has a 72% chance of requiring a tenotomy, which is done when the midfoot score is less than 0.5 [14].

This study aimed to assess a retrospective 5-years results of the Ponseti method in the treatment of congenital clubfoot which was performed for children aged under 2 years in western Amhara, Ethiopia, as a primary outcome and a descriptive analysis of factors affecting the outcome of the treatment.

## Objectives

### Primary

The objective of this study was to assess a 5-years results of the Ponseti method in the treatment of congenital clubfoot among under 2-years old children in Felege Hiwot Referral Hospital, Bahir-Dar, Ethiopia, from 2015 to 2019 G.C.

### Secondary

A descriptive analysis of factors affecting the outcome of the Ponseti treatment method.

## Methods

### Study design, period and settings

A retrospective study was conducted in Felege Hiwot Referral Hospital (FHRH), Orthopedic-Physio unit.

This study conducted in the Orthopedic Department in FHRH. FHRH is one of the biggest Specialized Referral Hospital in Amhara region and in the country at large. It was established by Emperor Haile Selassie in 1955 E.C/1963 G.C which landed in an area of 69,760 m square to primarily give services at a low cost or even free of charge to those who are unable to afford care elsewhere and at the time it was constructed to serve 20,000–25,000 people. The Hospital is currently serving 5–7 million people. It has been working as a medical college since 2000 E.C/2007G.C. Currently; it starts residency programs in six specialties in collaboration with Bahr Dar University, Collage of Medicine and Health Sciences (BDUCHS). The Hospital has more than 400 beds in all its wards and over 56 beds for orthopedic admissions per year.The study was conducted in Orthopedic-Physio unit. Ponseti treatment method started since 2013 G.C in FHRH but treatment started with its full capacity in May 1/2015 G.C. and around 500–1000 children with clubfeet were treated with Ponseti method during the study period.

### Source population

All children under 2-years old with a diagnosis of congenital clubfeet from May 1/2015 to April 31/2019 G.C who were admitted at Felege Hiwot Referral Hospital.

### Study population

All children under 2-years old with a diagnosis of congenital clubfeet from May 1/2015 to April 31 /2019 G.C and treated with Ponseti treatment method at Felege Hiwot Referral Hospital.

### Eligibility

#### Inclusion criteria

Children whose idiopathic clubfeet treated with Ponseti and children who started cast at age of 2 weeks.

#### Exclusion criteria

Children who were not seen by physiotherapy, discontinued their follow-up, with neurologic complication and children with syndrome clubfoot were excluded from the study.

#### Sample size and sampling technique

Assumptions *Z* statistic: The results of this study were presented with a level of confidence of 95%. So, the *Z* value is 1.96. Expected proportion (*p*): there was study on Ponseti treatment outcome at Mekelle University Hospital [16]. The Ponseti treatment success rate at Mekelle University Hospital was 77.9%. So, *P* of 0.779 was used to calculate the sample size. Precision (*d*): *d* = 0.05.

Sample size: $n = \frac{{Z^{2} p\left( {1 - p} \right)}}{{d^{2} }} = \frac{{1.96^{2} \; \times \;0.779\; \times \;\left( {1 - 0.779} \right)}}{{\left( {0.05*0.05} \right)}}\; = \;265$. Although the calculated sample size was 265, we only found 200 children’s charts with completed necessary data used for final analysis. A non-probability, convenience, sampling technique was used to review medical record of patients consecutively.

#### Data collection procedures

Data were collected by two trained data collectors (physiotherapists). Complete checklist and questionnaire were adopted from similar literatures modified based on the study context and as if it was convenient to collect the data from existing source. Parents of the child were involved for further information and complete the lost data from the existing source through their contacts. Two data collectors from Felege Hiwot Referral Hospital physiotherapy clinic were involved under close supervision by the investigators and questions regarding the study were clarified. Two chart room workers were participated to collect the charts from the store.

#### Study variables

##### Independent variables

Distance from facility, deformity pattern, residency, Pirani score, age, sex, family history of clubfoot, educational status of the parents, clubfoot type and religion.

##### Dependent variables

Ponseti treatment outcome.

#### Operational definitions

Clubfoot is said to be cured if the patient obtains cosmetically acceptable, pliable, functional, painless, plantigrade foot with pirani score one and 0 and not cured otherwise [16]. Pirani scoring system takes into consideration six different components of clubfoot deformity: posterior crease (PC), emptiness of the heel (EH), rigidity of equinus (RE), medial crease (MC), curvature of the lateral border (CLB) of the foot and reducibility of the lateral part of the head of the talus (LHT). Each component is scored on a three-point scale (0 = No abnormality, 0.5 = Moderate abnormality and 1 = Severe abnormality): the sum constitutes a total on a six-point scale (total score, TS), where higher scores indicate a more severe deformity. TS is divided into two subtotal scores, representing midfoot (midfoot score [MFS]: summing up MC, CLB and LHT) and hindfoot contracture (hindfoot score [HFS]: summing up PC, EH and RE), each ranging from 0 to 3 [17].

#### Clubfoot management in Felege Hiwot referral hospital

In Felege Hiwot Referral Hospital, clubfoot is managed in physiotherapy clinic on separated room from other patients. Physiotherapists are still the one who was responsible for assessing, appointing, casting and the overall management of clubfoot and one surgeon for performing tenotomy. The physiotherapists started the management with complete assessment on printed out format then continue for clinical judgments. They then educate parents for treatment technique and its compliances.

The clubfoot management primarily is based on the basic Ponseti principles. Patients are either casted or appointed based on the parents’ acceptability and stability after careful education about the technique at their first visit. Casting continues once a week till the physiotherapist decides the need for tenotomy or bracing. They apply the cast for the first, second, third, etc., visits to correct cavus, adductus and varus at variable number of casts according to the severity of the deformed position. Equinus is the last component of the deformity to be corrected with tenotomy or casting only. Tenotomy was conducted in the casting room by a single surgeon. Post tenotomy POP cast is applied for 3 weeks to ensure healing of the Achilles tendon. Once casting is finished, the patients are transferred to bracing or maintenance phase. Braces are prepared in the hospital by one technician. Parents are appointed to come 2 weeks after the first brace to check for compliances. The follow-up continues until the child starts to walk and get skeletal maturation. The appointments vary from 3 to 6 months and 1 year depending on the correction of the deformity.

#### Data quality assurance, processing and analysis

Checklist was properly designed and data collectors were well supervised by the investigators to ensure the data quality. The data collectors were trained for one day and they conducted pretest on 5% of the sample which were not included in the actual data. Every data sheet was checked and evaluated after collection for its completeness.

Statistical analysis was done using Statistical Package for Social Sciences software version 21 for windows (SPSS 21 Trademark of IBM Corporation). Descriptive statistics were performed and the results were presented with texts and tables.

#### Ethical clearance

Ethical clearance letter was obtained from Institutional Review Board (IRB) of Bahir Dar University, School of Medicine, and formal institution-based letter was sent to Felege Hiwot Referral Hospital for seeking cooperation and accordingly permission was obtained for the data collection. The data extracted from medical registration charts and every data was kept confidential by securing personal information in passwords, and name of patients were not used at all.

## Results

### Sociodemographic characteristics of the study participants and clinical characteristics

Out of 265 study participants, 200 included in the study with a response rate of 76%. The overall success rate of the Ponseti treatment method in Felege Hiwot Referral Hospital among 200 children with congenital clubfeet was 187 (93.5%) with a 95% (90–99.5). Sixty-five congenital clubfeet children were excluded from the study due to incomplete data. From the total respondents, majority of them 133 (66.5%) were males, 179 (89.5%) were Christian. Eighty-four (42%) were 70 km far from the Hospital (Table 1).

**Table 1:** Sociodemographic characteristics of study participants, (*N* = 200)

Variable	Frequency	Percentage (%)
*Gender*		
Male	133	66.5
female	67	33.5
*Age in month (s)*		
0–5	133	66.5
6–12	50	25
12–24	17	8.5
*Religion*		
Christian	179	89.5
Muslim	21	10.5
*Distance (in kilometer)*		
0–70	84	42
71–140	63	31.5
> 140	53	26.5

### Behavioral characteristics of parents

One hundred sixty-eight (84.0%) of the children’s mothers were housewife. Majority of the parents of the children (82.4%) had no formal education (Table 2).

**Table 2:** Behavioral characteristics of parents, (*N* = 200)

Variables	Frequency	Percentage (%)
*Educational status of the parents*		
No formal education	154	77
Primary school	25	12.5
Secondary school	13	6.5
College and above	8	4
*Occupation of the mothers*		
Housewife	168	84
Employed	31	15.5
Self-employed	1	0.5

### Birth related characteristics

From the study participants, 83 (41.5) of respondents born at home. Regarding birth related factors, majority of participants 165 (82.5) had no family history of clubfoot (Table 3).

**Table 3:** Birth related characteristics of the study participants, (*N* = 200)

Variables	Frequency	Percentage (%)
*Family history of clubfoot*		
Yes	35	17.5
No	165	82.5
*Complication at birth*		
Yes	200	100
No	0	0
*Place of birth*		
Home	83	41.5
Health center	84	42
Hospital	33	16.5
*Referral source*		
Self	25	12.5
Health center	82	41
Hospital	93	46.5

### Deformity severity

Among total participants, 143 (71.5%) had unilateral deformity and 57 (28.5%)) had bilateral deformity. Sixty-nine (34.5%) had 6.00 PIRANI score baseline. One hundred eighty-seven (93.5%) had 1–2 months duration of treatment (Table 4).

**Table 4:** Deformity severity of the study participants, (*N* = 200)

Variables	Frequency	Percentage (%)
*Site*		
Right foot	89	44.5
Left foot	54	27
Both feet	57	28.5
*Pattern of deformity*		
Unilateral	143	71.5
Bilateral	57	28.5
*Duration of treatment*		
1–2 months	187	93.5
3–4 months	12	6
> 5 months	1	0.5
*Pirani score*		
Pirani score = 6	69	34.5
Pirani score = 0–6	131	65.5

### Treatment factors

Among total participants, 162 (81%) were treated by cast with tenotomy brown splint and 12 (6%) participants treated with greater than six cast application. 189 (94.5%) of them had regular consultation with their orthopedician. One hundred ninety-six (98.0%) of the study participant had good adherence to brace (Table 5).

**Table 5:** Treatment factors to study participants, (*N* = 200)

Variables	Frequency	Percentage (%)
*Treatment methods*		
1–6 cast application	8	4
> 6 cast application	12	6
Cast with tenotomy brown splint	162	81
Referral to surgery	18	9
*Seeking consultation*		
Regular	189	94.5
Irregular	11	5.5
*History of previous treatment*		
Yes	5	2.5
No	195	97.5
*Treatment complications*		
Cast compression/slipped	2	1
No complication	198	99
*Brace compliance*		
Good	196	98
Poor	4	2

## Discussion

Clubfoot is one of the most prevalent musculoskeletal congenital defects [18]. The deformity includes four components: metatarsus adductus, cavus, hindfoot varus and equinus [3].

Major clubfoot surgery was not commonly indicated among patients treated with the Ponseti method. The Ponseti clubfoot technique has reduced total care costs, cast utilization, clubfoot surgery frequency and has also changed the patterns of surgery performed for clubfoot [19].

This study aimed to assess the 5-years results of the Ponseti method in the treatment of congenital clubfoot which was performed for children aged under 2 years in western Amhara, Ethiopia.

The overall success rate of the Ponseti method in the treatment of congenital clubfoot which was performed for children aged under 2 years in western Amhara, Ethiopia, among 200 children with clubfeet was 187 (93.5%) with a 95% (CI 90–99.5). The success rate of the current study was higher compared with a study conducted in Mekelle which revealed that 77.9% [16]. This could be justified with lack of emphasis for the Ponseti method in the previous study as authors stated. But, similar with a systematic review that explored Ponseti method in the management of clubfoot under 2 years of age [20]. This could be the fact that currently Ponseti method for the treatment of clubfeet is the gold standard and practiced in the world. However, the current study was higher than a study conducted in Nigeria showed that a painless plantigrade foot was obtained in 255 feet (78%) without the need for extensive soft tissue release and/or bony procedures [21]. The reason might be their study was a retrospective study to evaluate the efficacy of the Ponseti method for initial correction of untreated, idiopathic clubfoot in patients above 1 year of age, but ours include below 2 years, which might be supported that early treatment revealed better outcome.

In the other way, it was lower than a study performed by Matar HE, et al. showed that initial correction was achieved in all children with an average of 8 (range 4–10) Ponseti casts [22]. This discrepancy might be the current study reviewed only the 5-years’ charts but their study review over 10 years retrospectively.

In this study 77% of parents of the study participants had no formal education, 41.5% were born at home, 2% had poor bracing compliance. These factors might have negative role for the success rate of the treatment method.

This finding was supported a study which revealed that non-compliance in seventeen patients (17.7%) with post tenotomy bracing, educational level (less than high school) of parents (36.7%) with odds ratio of 5.5 (*P* = 0.0073) and access of parents to internet (Yes or No) were some of the independent factors attributes of the patients associated with poor outcome after use of Ponseti method in idiopathic clubfoot management [10].

In this study, males were almost 2 × affected by the disease (clubfoot). It is well studied that idiopathic clubfoot is approximately twice as common in males as in females. The reason for this discrepancy is unclear but may represent an inherent difference in the susceptibility to the deformity [23].

In the current study, 65.5% of the children with clubfeet had a Pirani score of 0–6. The severity of the disease and effectiveness of the treatment was also assessed by the Pirani score. This score was effective for assessment of the severity of the deformity and the measurement of the outcome of the Ponseti treatment [12, 14, 24]. The current study showed that 81% of the total participants of the study were referred for tenotomy and the procedure was conducted necessarily on both sides for bilateral cases. This figure is higher compared to other studies [4, 5, 8, 18–21, 24]. It can be reasoned out that the current study included higher sample size and duration.

## Limitations of the study

The facility did not utilize a constant patient assessment format which leads to poor quality of registration and reporting that is why 65 patients’ charts were excluded from the study.

This is only a descriptive study that did not indicate the association of factors to outcome with appropriate analysis method.

## Conclusions and recommendations

According to the results from a 5-years data showed that the Ponseti method in the treatment of congenital clubfoot was successful with a success rate of 93.5%. We recommend that children with congenital clubfeet should be managed with Ponseti treatment method timely.
